# Dynamics of the dosage of citrate-calciuminfusion regimens as anticoagulation in continuous venovenous hemodiafiltration

**DOI:** 10.1186/2197-425X-3-S1-A56

**Published:** 2015-10-01

**Authors:** H Marin Mateos, JA Barea Mendoza, S Chacon Alves, G Morales Varas, I Saez de la Fuente, R Garcia Gigorro, JA Sanchez-Izquierdo Riera, JC Montejo González

**Affiliations:** Hospital 12 de Octubre, Madrid, Spain; Hospital 12 de Octubre, Medicina Intensiva, Madrid, Spain

## Objectives

To describe the dynamics of the infusion regimen 's dosage of the Citrate-Calcium, when they are used as anticoagulants in systems of CRRT and, determine if the numbers of changes reduce and reach a constant velocity of infusion as the therapy times progresses, to prove the utility of dynamic dosage in hemodiafiltration therapy.

## Methods

A descriptive and comparative study about a prospective data basis is made. All the patients with CRRT requirements, admitted in ICU from September 2014 to March 2015 are included. Variables as motive of therapy, daily dosage of the therapy, days of therapy, half-life of systems on the first three days, global half-life of systems, type of anticoagulation, daily mean dosage of citrate-calcium, number of daily changes in these dosages and complications are analysed. The number of changes of citrate-calcium and the day of therapy is compared, such as the filter's length and the kind of anticoagulation.

## Results

A total of 36 patients are obtained, with a therapy's length of 331 days, an average of 7.31 days (SD 7.10), a mean half-life system on the first three days of 2.13 days (SD 0.9), a mean dialysis dosage 1411 ml/h (SD 494.6), an average filtration dosage 700.9 ml/h (SD 583.1), removal dosage 99.8 ml/h (SD 71.5). Indication of therapy was management of volume 61% and filtratrion of Urea and electrolytes in 33% of the cases. The type of the anticoagulation was Heparine 61% and Citrate-Calcium 25%.

A total of 62 days of anticoguation with citrate is described, the average of length 6.8 days. Mean Citrate dosage was 3.02 mmol/l (SD 0.47) and mean calcium dosage 1.5mmol/l (SD 1.04). No significant bleeding, severe electrolyte, or calcium disorders were observed.

A progressive decrease of dialysis and convection dosage is observed along the days of treatment with linear regression (p < 0.01). Consequently a decrease of 25% of dyalisis solutions consumption is noticed compared to historical controls.

Global half-life systems were longer in patients with Citrate-Calcium regimens, average 67.25 hours (IC 60.33-74.16) compared to patients with Heparine 54.14 hours IC(47.47- 60.80) p < 0.05.

Also is described a reduce in the number of changes in the citrate-calcium dosage along the period of therapy correlation coefficient (p < 0.05).

## Conclusions

A dynamic dose of hemodiafiltration is taken in our ICU patients with CRRT requirements, consequently a greater efficiency in therapies is observed. Changes in Citrate-Calcium dosage descreases along the days and reaches a constant velociy infusion, so Citrate-Calcium regimens becomes a safe and easy-managment option for anticoagulation in critical patients.

